# Supplementary material for: Glycyrrhetinic Acid Improves Insulin-Response Pathway by Regulating the Balance between the Ras/MAPK and PI3K/Akt Pathways
Source: Nutrients. 2019 Mar 12;11(3):604. doi: 10.3390/nu11030604 (PMC6470473; doi:10.3390/nu11030604)

Supporting information

Glycyrrhetinic acid improves insulin resistance by regulating the balance between the Ras/MAPK and PI3K/Akt pathways

Yuan Zhang[1], Shengnan Yang[1], Man Zhang[1], Zhihua Wang[1], Xin He[1], Yuanyuan Hou[1*], Gang Bai[1*]

E-mail address: houyy@nankai.edu.cn and gangbai@nankai.edu.cn

1. Abbreviations

DMEM: Dulbecco's modified eagle medium

NH_2-MMs: Fe_3O_4 amino magnetic microspheres

EDCI:1-[3-(Dimethylamino)propyl]-3-ethylcarbodiimide hydrochloride

HOBt : Nhydroxybenzotrizole

Sulfo-SADP: [sodium1-((3-((4-azidophenyl)disulfanyl)propanoyl)oxy)-2,5-dioxopyrrolidine-3-sulfonate]

N_3-tag: 3-azido-7-hydroxy-2H-chromen-2-one

FBS: Fetal bovine serum

PBS: Phosphate-buffered saline

PAGE: Polyacrylamide gel electrophoresis

SDS: Sodium dodecyl sulfate

DTT: DL-dithiothreitol

2. Target prediction of GA

To screen the potential target proteins of GA, the top 20 protein targets (determined based on fit value using PharmMapper were software) were analyzed using bioinformatics tools. Next, the interacting proteins were analyzed using String 10.0 (http://www.string-db.org/). We used AutoDock 4.2 software to conduct molecular docking studies to evaluate the interaction targets and GA. At last, we performed capture assays to verification of target proteins.

Reverse docking of GA was performed using the PharmMapper server (http://59.78.96.61/pharmmapper/). We prepared a 3D structure of GA in the sdf format using the ChemBio3D Ultra 13.0 software (PerkinElmer Inc., U.S.A.), and submitted the sdf file to the PharmMapper server with the choice of "human protein targets only" and the maximum generated conformations set to 300. In our study, we employed the 2010 version. The first 20 candidate targets of GA are listed in (Supplementary Table S1). Next, the interacting proteins were analyzed using String 10.0 (http://www.string-db.org/). Three targets in the insulin-related signaling pathways, namely, HRAS, PRKCA (PKCα) and MAP2K1 (MEK1). In addition, two targets in the steroid hormone biosynthesis signaling pathways, HSD11B1 and HSD17B1

The three-dimensional structures of the MAP2K1, PKCα , HRas, HSD11B1 and HSD17B1 proteins (PDB ID code 4U81, 3IW4, 4EFL, 3CZR and 3HB4) were obtained from the Protein Data Bank (http://www.rcsb.org/pdb). The structures of the MAP2K1, PKCα, HRas, HSD11B1 and HSD17B1 proteins were constructed and minimized using the SYBYL software (Chemical Computing Group, Inc.); then, AutoDock version 4.2 (Olson Laboratory, La Jolla, CA) was applied to perform a docking study using a hybrid Lamarckian genetic algorithm (LGA). The number of LGA runs was set to 30. The step size parameters of quaternion and torsion were 30. The binding energies of the GA target proteins HRAS, PKCα, MEK1, HSD11B1 and HSD17B1 were -7.47, -8.77, -9.93, -10.31 and -10.12 kcal/mol kcal/mol, kcal/mol, respectively.

Table S1. Top 20 proteins output from prediction of PharmMapper server.

No.	Fit	Num Feature	Name
1	6.737	10	Corticosteroid 11-beta-dehydrogenase isozyme 1
2	5.537	10	Transthyretin
3	5.083	7	Amine oxidase [flavin-containing] B
4	4.862	10	Cellular retinoic acid-binding protein 2
5	4.788	8	Retinol-binding protein
6	4.660	9	Dihydroorotate dehydrogenase (quinone), mitochondrial
7	4.630	15	GTPase HRas
8	4.606	10	Retinoic acid receptor RXR-beta
9	4.581	16	Estradiol 17-beta-dehydrogenase 1
10	4.562	13	Vitamin D3 receptor
11	4.554	9	Dual specificity mitogen-activated protein kinase kinase 1
12	4.459	12	Alpha-tocopherol transfer protein
13	4.412	7	Lanosterol synthase
14	4.400	11	Methionine aminopeptidase 2
15	4.335	6	Glutathione S-transferase A1
16	4.333	8	Androgen receptor
17	4.297	10	Retinoic acid receptor gamma
18	4.281	10	Nuclear receptor subfamily 1 group I member 2
19	4.251	8	Retinoic acid receptor RXR-alpha
20	4.175	9	Protein kinase C alpha type

3. Synthesis section

General Chemical Reagents and Methods

All reagents purchased for synthesis. Thin-layer chromatography (TLC) was performed on silica gel GF254 plates with detection using shortwave UV light (λ=254 nm) and staining with 10% phosphomolybdic acid in EtOH, followed by heating on a hotplate. Flash chromatography was performed with silica gel (100-200 mesh) with EtOAc/petroleum ether or CH_2Cl_2/MeOH as eluent. ^1H and ^{13}C NMR spectra were recorded on a Bruker AV 400 spectrometer at 400 MHz (^1H NMR) and 100 MHz (^{13}C NMR), using $CDCl_3$ as solvents. Coupling constants are displayed in Hertz.

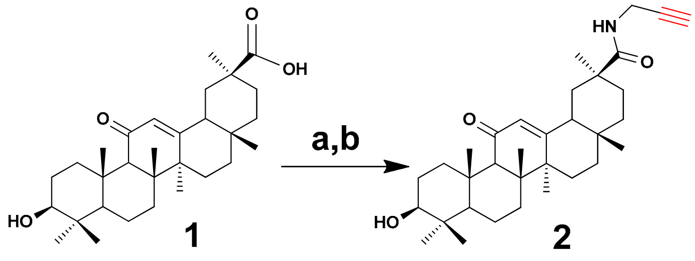

Figure S1. Synthetic route for **compound 2** (Alkynyl-GA). Reagents and conditions: (a) EDCI, HOBt, triethylamine, dichloromethane; (b) mono-Propargylamine, dichloromethane.

Compound 2 Synthesis (Alkynyl-GA)

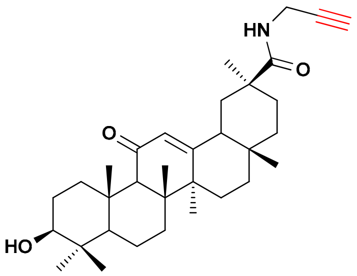

To a solution of **compound 1** (0.9414 g, 2.0 mmol) in 20 ml of dichloromethane. EDCI (0.4601g, 2.4mmol), HOBt (0.3243 g, 2.4 mmol) was added. The mixture was stirred for 0.5 h at 0 °C. Then triethylamine (8.0 mmol, 1115 μL) was added dropwise and stirred for 0.5 h at 0 °C. Then mono-propargylamine (3.0 mmol, 206 μL) was added and the mixture was stirred for 1 h at 0 °C and stirred at room temperature for 2 days. The reaction was quenched with water and extracted with dichloromethane (3×25 mL). The combined organic layers were dried over Na_2SO_4, and concentrated to give a crude product. The crude product was solubled in 3 mL methylene chloride and purified by column chromatography on silica gel (dichloromethane: methanol = 30:1) to get the desired products **compound 2**, a white solid (0.4814 g，47.8%) (Figure S3). ^{1}H NMR (400 MHz, $CDCl_3$) δ 5.83 (s, 1H), 5.66 (s, 1H), 4.04 (dd, *J* = 39.7, 17.6 Hz, 2H), 3.21 (d, *J* = 5.4 Hz, 1H), 2.76 (d, *J* = 12.9 Hz, 1H), 2.27 (d, *J* = 33.0 Hz, 2H), 2.12 (s, 1H), 2.00 (d, *J* = 14.6 Hz, 1H), 1.91 (d, *J* = 9.3 Hz, 1H), 1.86 – 1.70 (m, 3H), 1.58 (d, *J* = 13.1 Hz, 4H), 1.52 – 1.30 (m, 9H), 1.27 – 1.07 (m, 10H), 1.05 – 0.88 (m, 5H), 0.79 (s, 5H), 0.67 (d, *J* = 11.5 Hz, 1H). ^{13}C NMR (100 MHz, $CDCl_3$) δ 200.4, 175.7, 169.3, 128.8, 79.9, 79.0, 71.9, 62.0, 55.1, 48.2, 45.6, 43.7, 43.4, 42.0, 39.3, 37.5, 37.3, 32.9, 32.1, 31.6, 29.5, 28.6, 28.3, 27.5, 26.7, 26.6, 23.6, 18.9, 17.7, 16.6, 15.8 (Figure S2). HRMS (ESI$^+$): m/z $[M+Na]^+$ calculated for C33H49O3: 507.75; found: 530.3608. (Figure S3).

a

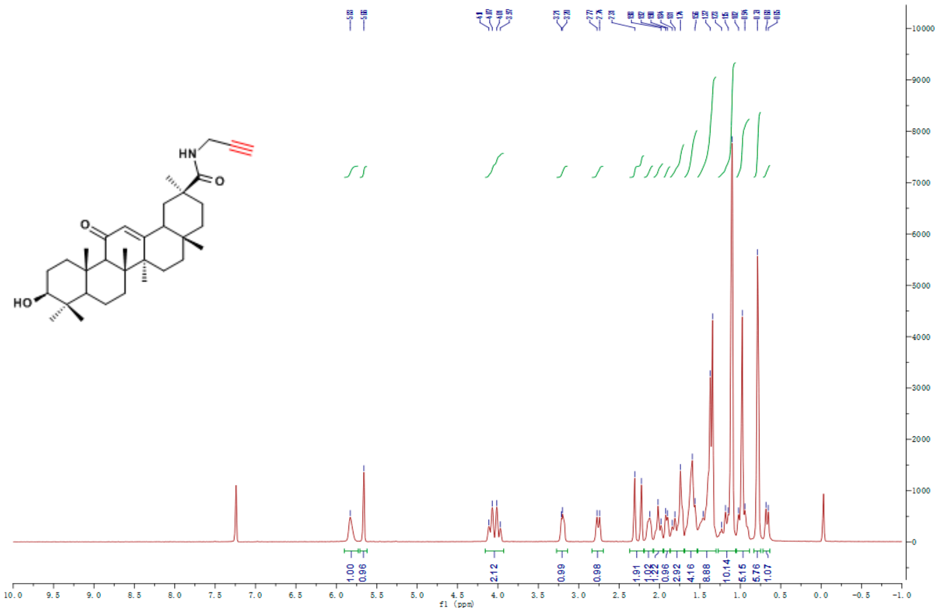

b

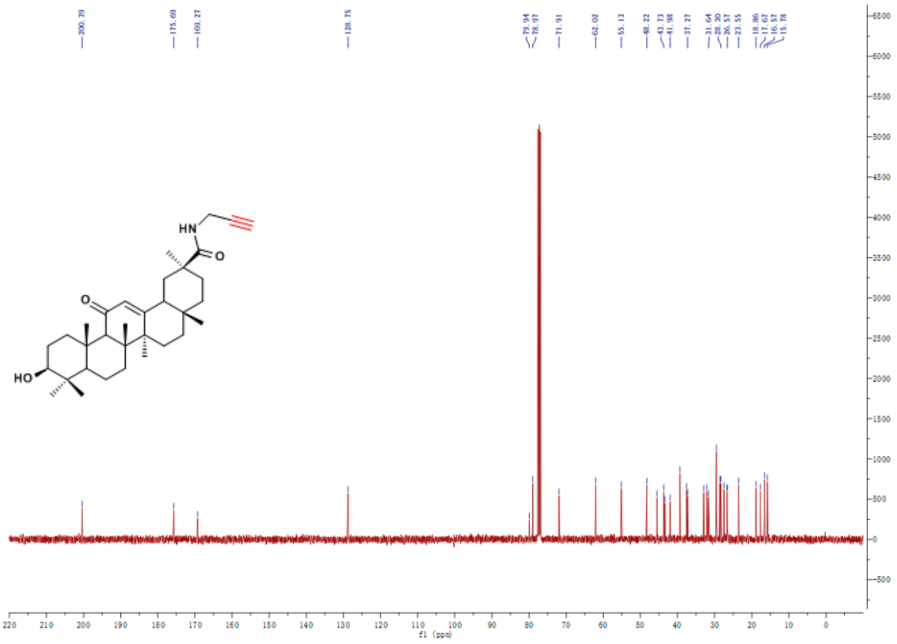

Figure S2. The NMR data of Alkynyl-GA, **(a)** ^{1}H NMR spectrum of Alkynyl-GA probe (400 MHz, $CDCl_3$) and **(b)** ^{13}C NMR spectrum of Alkynyl-GA probe (100 MHz, $CDCl_3$).

a

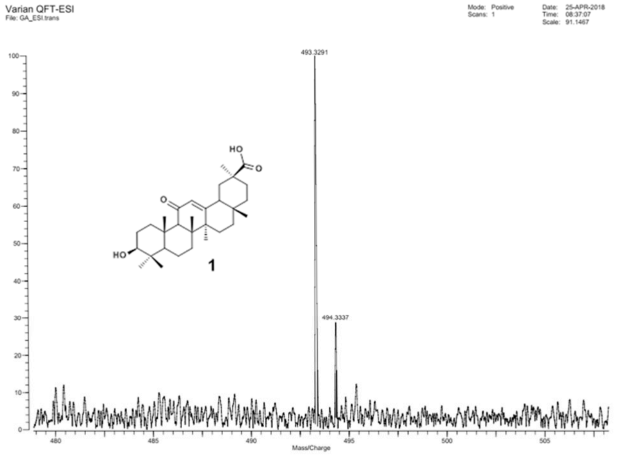

b

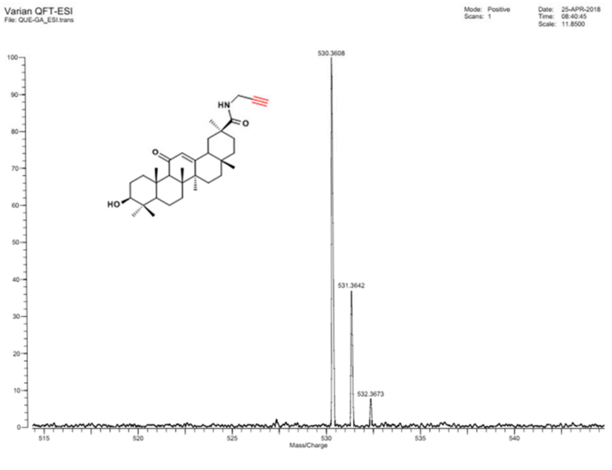

Figure S3. The HRMS data for alkynyl-GA. (a) The spectra of GA and (b) alkynyl-GA.

Probe1 characterization section

Fe_3O_4 amino magnetic microspheres (NH_2-MMs) were purchased from Tianjin baseline chromtech research centre (Tianjin, China). The synthetic route for **Probe1** was shown in (Figure S4).

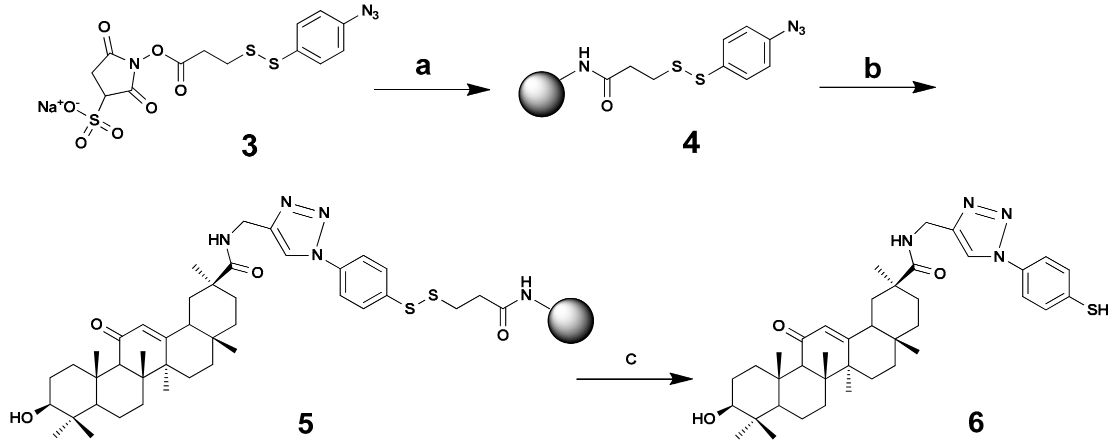

Figure S4. Synthetic route for **Probe 1** (**compound 5**). Reagents and conditions: (a) MMs-NH2, borate buffer, DMSO; (b) CuBr, DIPEA, methanol; (c) DTT, methanol.

Azide modified-MMs Synthesis (Compound 4)

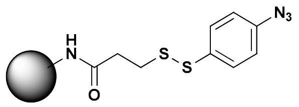

5 mL NH2-MMs (25 mg, 5 mg/mL) were suspended in 2 mL borate buffer and Sulfo-SADP (**compound 3**) (0.5 mg, 11 μmol) were added in sequence, the mixture was shocked at room temperature for 12 h. After that, the azide modified-MMs was enriched through magnetic separation and washed with water for three times. The gathered azide modified-MMs **(Compound 4)** was used directly for next steps.

Probe 1 (Compound 5)

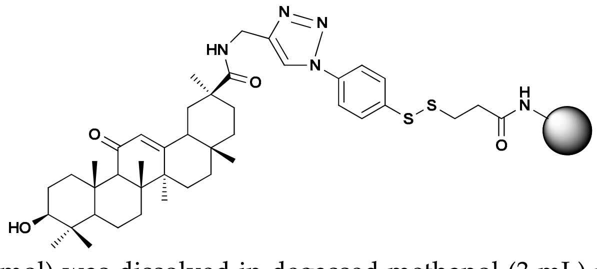

CuBr (10 mg, 0.7 mmol) was dissolved in degassed methanol (3 mL) under argon atmosphere and DIPEA (35 μL, 0.18 mmol) was added. The resulting yellowish suspension was degassed for 30 min under a stream of argon and with exclusion of light. Alkynyl-modified GA probe **compound 2** (5 mg, 10 μmol) was dissolved in degassed methanol (0.5 mL) and treated with 1 mL of the freshly prepared suspension of CuBr-DIPEA and azide modified-MMs (25 mg, 5 mg/mL). The reaction mixture was shocked at room temperaturewith exclusion of light for 24 h. Then the GA-modified functionalized MMs were separated with magnet and washed three times each with methanol and water. The gathered GA-modified functionalized MMs (**Compound 5**) was used directly for next steps.

Compound 6

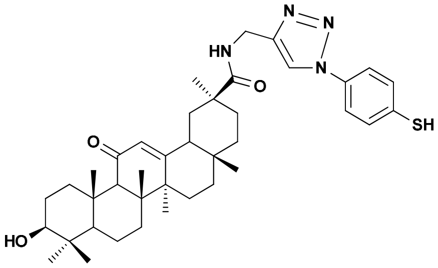

To a solution of **probe1**, **probe1** (25 mg, 5 mg/mL) in 1 mL methanol, DTT (100 mM) were added. The reaction mixture was shocked at room temperature for 1h. Then the was removed the MMs with magnet and get **compound 6**

Probe 2 characterization section in (Figure S5)

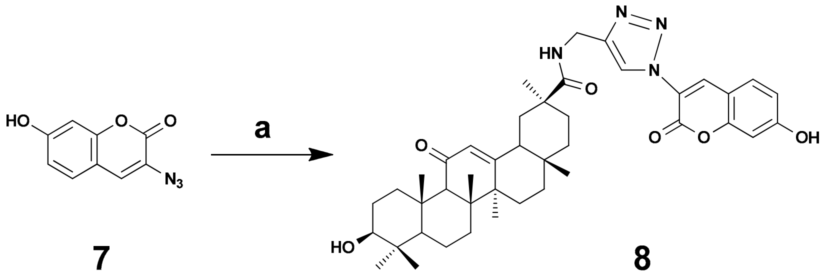

Figure S5. Synthetic route for click chemistry product **probe2** (**compound** 8). Reagents and conditions: (a) CuSO4, sodium ascorbic acid, DMSO, Alkynyl-GA.

Compound 7 (N_3-tag)

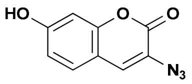

The synthesis of **Compound** 7, N_3-tag, (3-azido-7-hydroxy-2H-chromen-2-one) was delegated to Wuxi App Tec (Beijing, China).

Compound 8 (probe 2)

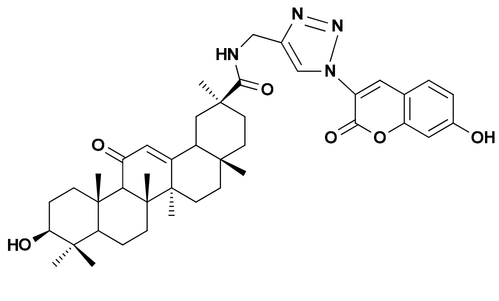

Supplement: Supplementary file 1 [file nutrients-11-00604-s001.pdf]
